# Post‐eclosion temperature effects on insect cuticular hydrocarbon profiles

**DOI:** 10.1002/ece3.7050

**Published:** 2020-12-07

**Authors:** Subhash Rajpurohit, Vladimír Vrkoslav, Robert Hanus, Allen G. Gibbs, Josef Cvačka, Paul S Schmidt

**Affiliations:** ^1^ Division of Biological and Life Sciences School of Arts and Sciences Ahmedabad University Ahmedabad India; ^2^ Department of Biology University of Pennsylvania Philadelphia PA USA; ^3^ Institute of Organic Chemistry and Biochemistry AS CR Prague Czech Republic; ^4^ School of Life Sciences University of Nevada Las Vegas NV USA

**Keywords:** cuticular hydrocarbons, desiccation tolerance, *Drosophila melanogaster*, eclosion, natural populations, phenotypic plasticity, water loss rate

## Abstract

The insect cuticle is the interface between internal homeostasis and the often harsh external environment. Cuticular hydrocarbons (CHCs) are key constituents of this hard cuticle and are associated with a variety of functions including stress response and communication. CHC production and deposition on the insect cuticle vary among natural populations and are affected by developmental temperature; however, little is known about CHC plasticity in response to the environment experienced following eclosion, during which time the insect cuticle undergoes several crucial changes. We targeted this crucial to important phase and studied post‐eclosion temperature effects on CHC profiles in two natural populations of *Drosophila melanogaster*. A forty‐eight hour post‐eclosion exposure to three different temperatures (18, 25, and 30°C) significantly affected CHCs in both ancestral African and more recently derived North American populations of *D. melanogaster*. A clear shift from shorter to longer CHCs chain length was observed with increasing temperature, and the effects of post‐eclosion temperature varied across populations and between sexes. The quantitative differences in CHCs were associated with variation in desiccation tolerance among populations. Surprisingly, we did not detect any significant differences in water loss rate between African and North American populations. Overall, our results demonstrate strong genetic and plasticity effects in CHC profiles in response to environmental temperatures experienced at the adult stage as well as associations with desiccation tolerance, which is crucial in understanding holometabolan responses to stress.

## Graphical abstract

This work compares cuticular hydrocarbon profiles of ancestral African *D. melanogaster* populations with North American *D. melanogaster* populations. We report huge differences in cuticular hydrocarbon profiles between ancestral and recently migrated (within 300 years) populations of *D. melanogaster*. We employ various temperature manipulation approaches to understand cuticular hydrocarbons fate. Later, we try to understand cuticular hydrocarbons role in physiological processes, that is, desiccation tolerance/water loss rates.

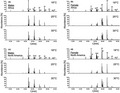

## 1 INTRODUCTION

The insect cuticle is a complex biological structure (Chapman, 2013). The outermost layer is made up of long‐chain hydrocarbons (mainly alkanes, alkenes, and branched alkanes) produced by specialized cells known as oenocytes and subsequently transported to the insect cuticle (Makki et al., 2014). One role of these cuticular hydrocarbons (CHCs) is in controlling water loss through the cuticle (Hadley, 1994; Jallon & David, 1987; Gibbs, 1998; Gibbs, 2002a, 2002b; Gibbs & Rajpurohit, 2010). Insects lose >70% of their body water though the cuticle and the deposition of CHCs has been proposed as a potential mechanism to conserve water (Gibbs, 1998; Gibbs, 2002a, 2002b; Gibbs & Rajpurohit, 2010). It has been shown experimentally that removal of CHCs by organic solvents such as hexane causes an increase in the rate of desiccation (Wigglesworth, 1945). In addition to potential function in the stress response, CHCs are also involved in pheromonal and other chemical communications (Bagneres et al., 1996; Berson et al., 2019; Blomquist & Bagneres, 2010; Chung & Carroll, 2015; Fan et al., 2013; Kuo et al., 2012; Le Conte & Hefetz, 2008; Oystaeyen et al., 2014). Geographical variation in these compounds has also been reported in many *Drosophila* species from Australia and North America (Etges et al., 2017; Francesca & Chenoweth, 2010; Matzkin et al., 2007; Rajpurohit et al., 2013, 2017).

The length and structure of CHCs are associated with distinct functional properties. Longer chain length (number of carbon atoms) corresponds to a higher melting point (Gibbs & Pomonis, 1995) and potentially greater waterproofing (Rourke & Gibbs, 1999). The presence of double bonds and/or branching structures is associated with lower melting point but greater information content for chemical communication (Blomquist & Bagneres, 2010). It has been suggested that melting temperatures of CHCs are directly correlated with waterproofing properties but inversely correlated with information content (see Figure 2 in Chung & Carroll, 2015). Thus, CHC profiles in natural populations may represent a functional trade‐off and evolve in response to environmental conditions that vary spatially and/or temporally. While CHC profiles have a genetic basis (Dembeck et al., 2015; Rajpurohit et al., 2017), they also exhibit pronounced plasticity in response to developmental temperature (Rajpurohit et al., 2017). Thus, understanding the relative roles of genetic variation, plasticity, and genotype by environment interactions in the production and functional effects of CHC profiles is a major goal in understanding the dynamics of stress resistance in natural populations. Natural habitats are heterogeneous and situate organisms to handle diverse environmental conditions. The dynamic nature of CHC profiles could respond to these environmental perturbations and play a significant role in molding life‐history traits.

Eclosion is the process in which holometabolous insects emerge from the pupal case. Newly eclosed adults normally expand their wings (Hari et al., 2008) and display tanning and hardening of their cuticle (Dewey et al., 2004). This is a very crucial transition from a nonmoving life stage to a free life stage. The newly eclosed adult is relatively vulnerable to predation until its cuticle hardens, and is susceptible to water loss through the un‐tanned cuticle. Water loss is temperature sensitive (Gibbs & Rajpurohit, 2010), so temperature immediately after eclosion can have significant effects on water balance and survival. Numerous studies have examined the effects of temperature on CHC and water loss of drosophilids (Etges et al., 2017; Toolson, 1982, Gibbs et al., 1998; Rajpurohit et al., 2017; Krupp et al., 2019) and other terrestrial arthropods (Hadley, 1978; Toolson & Hadley, 1979; Rourke, 2000). In all cases, however, experimental animals were fully mature adults.

In this study, we investigate how temperatures affect CHC immediately post‐eclosion, while the cuticle is still tanning and hardening. We transferred *Drosophila melanogaster* adults immediately after eclosion to three different temperatures (18, 25, 30°C). *D. melanogaster* viable thermal window is between 12–32°C. The range picked by us (from 18–30°C) covers the entire viable thermal range of this species (see Hoffmann, 2010). After 48 hr, when their cuticles had darkened, we analyzed CHC composition, desiccation tolerance, and physiological parameters related to water balance.

## 2 MATERIAL AND METHODS

### 2.1 Stocks and population cages

To establish population cages we used 40 African (Victoria Falls, Zimbabwe; collected by B. Ballard) and 40 North American (Pennsylvania, USA; collected by Paul Schmidt) isofemale lines collected in 2010 and 2012 respectively. Two replicate cages were established for each geographical region using 20 independent lines per population per cage. Cages (1ft^3^, Bioquip product 1466AV) were founded with ten females per line (10 females x20 independent lines = 200 females) and were cultured at 25°C, 12L:12D in a Percival I36VL incubator. Lines were outcrossed for 5 generations prior to experimental use and cages were maintained at a census size of ~5,000 individuals.

### 2.2 Sample collection for cuticular hydrocarbon isolation

**Figure 1:**
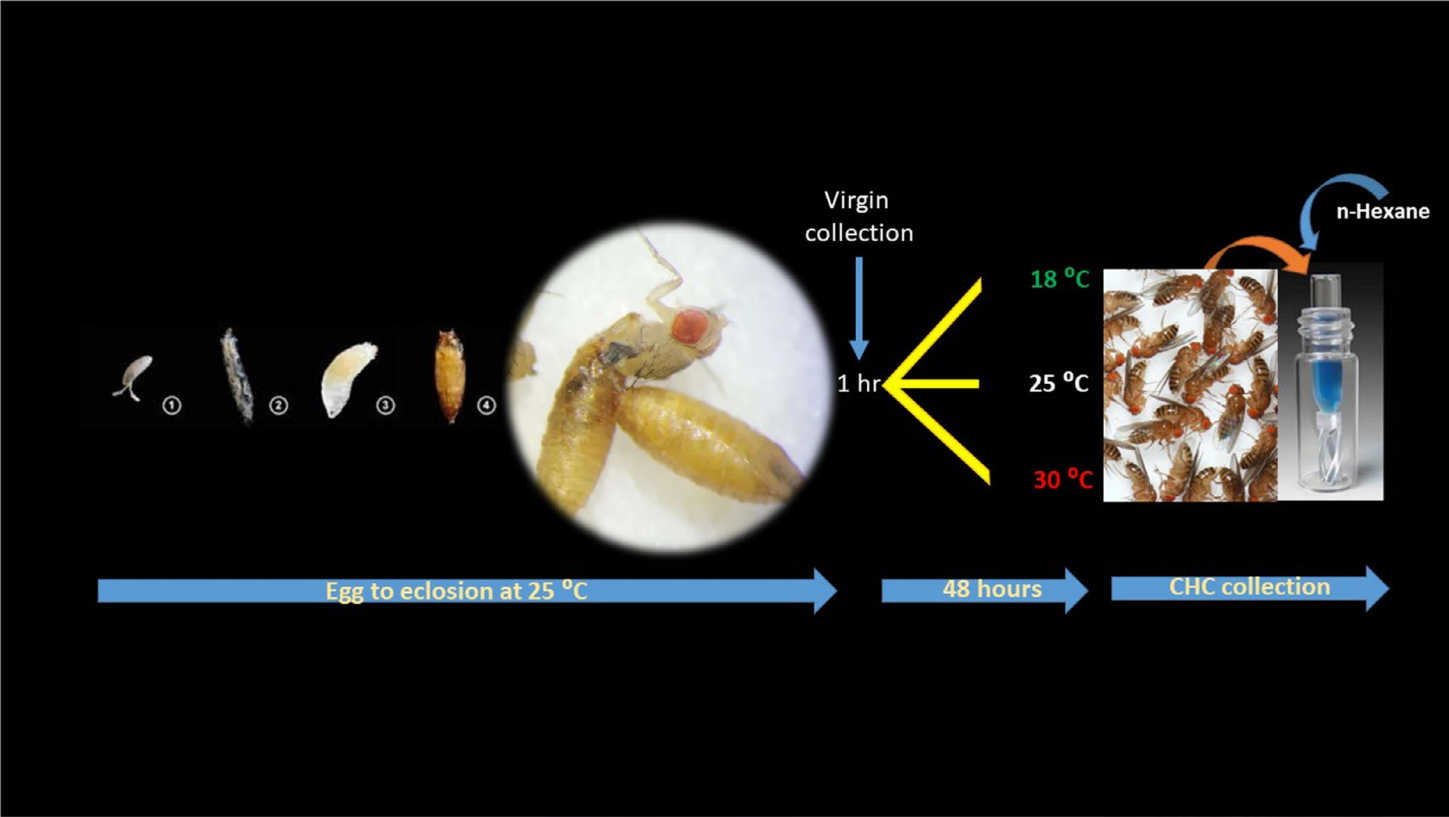
Schematic representation of overall experimental design and hydrocarbon collection process from *Drosophila melanogaster* adults exposed to three different temperatures (18, 25, and 30°C) for 48 hr. *Drosophila melanogaster* developmental stages and a fly emerging from its pupal case is also shown

Flies were allowed to lay eggs at room temperature (25°C) in bottles for 3–4 hr and transferred at low density in vials (30–40 eggs per vial). These vials were immediately moved to the 25°C incubator. On emergence, flies were collected within 1 hr, sexed, and immediately transferred to one of three temperature treatments (18, 25, and 30°C, all at 12L:12D in Percival I36VL incubators). For each temperature treatment, we collected 50 males and 50 females in triplicate (for both the continental populations). After 48 hr, post‐eclosion temperature treated flies were immediately transferred to empty glass vials and placed at −80°C. The overall experimental design is shown in Figure 1.

### 2.3 Isolation and purification of cuticular hydrocarbons

Dried *D. melanogaster adults* (three replicate groups of 50 individuals per population and treatment) were vortexed three times with 200 μl of hexane. After each addition of hexane, samples were vortexed for 60s. The extracts were then purified in 3 mm diameter columns, which were sealed at the bottom with cleaned cotton wool and filled with 75 mg of silicagel (particle size 50–100 μm). CHCs were eluted from the columns by addition of 3 × 100 μl volumes of hexane. Solutions were concentrated under a stream of nitrogen to approximately 25 μl and stored at −80°C.

### 2.4 GC‐MS analyses of cuticular hydrocarbons

GC‐MS analyses were performed using a 7890A gas chromatograph coupled with a 5975C mass spectrometer, equipped with electron ionization (EI) and quadrupole analyzer (Agilent Technologies, Santa Clara, CA, USA). The samples (1 μl) were injected to split/splitless inlet in split mode (split ratio 20:1). The injector temperature was 320°C. A DB‐1ms fused silica capillary column (30 m × 250 μm; a film thickness of 0.25 μm, J&W Scientific) was used for separation. The carrier gas was helium at a constant flow rate of 1.0 ml/min. The temperature program was: 50°C (1 min), then 50°C/min to 150°C, followed by 5°C/min to 330°C (10 min). The temperatures of the transfer line, ion source and quadrupole were 320°C, 230°C and 150°C, respectively. EI spectra (70 eV) were recorded from 25 to 600 m/z.

### 2.5 Cuticular hydrocarbon data mining

Peak areas of the GC chromatograms corresponding to individual CHCs were converted into relative proportions of the sum of CHC peak areas. Only the variables (CHCs) representing at least 0.5 relative percent in at least one individual of the given sample set were selected for further analysis. The relative proportions were then calculated again including only the selected peaks. The relative data were arcsine transformed and the overall quantitative variability among samples and treatments visualized using principal component analysis (PCA) performed in Statistica 8.0. Subsequently, the same relative and arcsine transformed data were used for canonical correspondence analysis (CCA) in Canoco 4.5, with intersample distance being analyzed using Hill's scaling followed by Monte Carlo permutation test with 999 permutations to assess the statistical significance of the differences among treatments. The reported *p* values for the Monte Carlo permutation tests were calculated from the formula *p* = (*n_x_* + 1)/(*N* + 1) where *n_x_* stands for the number of permutations with multivariate pseudo F‐ratio value equal or larger than that of the nonpermuted real dataset, and *N* represents the total number of permutations (Šmilauer & Leps, 2014). The variables represented by the relative percentages of individual CHCs fitting the best the calculated model of intertreatment differences were then extracted from the CCA and listed using CanoDraw plugin under Canoco 4.5.

### 2.6 Wet mass, dry mass, and water content

In the groups of five, forty‐eight hour old flies were weighed on a Sartorius microbalance (Gemplus Series CPA2250; Goettingen, Germany). To estimate the weight, the flies were frozen at −20°C and weighed immediately after removal from the freezer. All samples were measured within two‐three days of collection to avoid freezing‐associated dehydration. Dry weights were measured as the weight after drying at 50°C overnight. Total body water content was estimated as the difference between masses before and after drying at 50°C. As these data were collected from the subset of flies that had undergone 48 hr of temperature exposure in the experimental manipulation, we used temperature treatment as a covariate to rule out the differences in desiccation tolerance are not influenced by the initial wet mass/water content of the flies. The wet mass, dry mass, and water content values were used to generate residuals for ANOVA analysis of desiccation tolerance.

### 2.7 Desiccation tolerance

Four‐to‐five day old virgin flies were transferred to empty vials in groups of ten and restricted to the lower half of the vials by a foam stopper. Silica gel was then added above the stopper to maintain low humidity, and the vial was sealed with Parafilm™. Vials were subsequently transferred to a 25°C incubator and mortality was recorded at hourly intervals until all flies were dead. Five replicate vials per treatment were setup for desiccation assay. Each tube contained 5 individuals. We ran a mixed model ANOVA where population cages were nested within geographic source population.

### 2.8 Respirometry

Water loss rate (WLR) was measured using flow‐through respirometry (TR‐2 respirometer; Sable Systems, Las Vegas, Nevada, USA). Groups of 10–20 flies were placed in 5 ml glass/aluminum chambers, and dry CO_2_‐free air was pumped through the chambers at a flow rate of 50 ml/min to an LI‐6262 infrared CO_2_ sensor (Li‐Cor Biosciences, Lincoln, Nebraska, USA). Recordings began approximately 90 min after placement in the respirometer. Water loss rate was calculated from water vapor released by flies into the air stream. The humidity sensor was calibrated by injection of small drops of water (0.5–3.0 nl) into the air stream. Datacan V software (Sable Systems, NV, USA) was used for data collection and analysis. We ran a mixed model ANOVA where lines were nested within geographic source population.

## 3 RESULTS

Based on the retention time GC/MS showed 48 peaks for *D. melanogaster* CHCs. This cocktail of CHCs included single chain, branched, and double bond compounds (Table 1). We found a significant overlap with the compounds found in other CHC studies of *D. melanogaster* (see Dembeck et al., 2015 and Rajpurohit et al., 2017).

**Table 1:** *Drosophila melanogaster* CHCs identified by GC/MS. CHCs are denoted as the number of carbons:number of double bonds; “(br)” stands for branched

Peak number	**Rt [min]**	**CHC**	**Sex**	Peak number	**Rt [min]**	**CHC**	**Sex**
1	10.06	C17:0	♀♂	25	25.58	C27:0 br	♀♂
2	16.76	C21:0	♀♂	26	25.67	C27:2 + C27:1	♀♂
3	18.05	C22:1	♂	27	25.78	C27:1	♀♂
4	18.43	C22:0	♂	28	25.92	C27:1	♀
5	19.30	C23:2	♀	29	26.07	C27:0	♀♂
6	19.48	C23:0 br	♀♂	30	26.94	C28:0 br	♀
7	19.58	C23:1	♀♂	31	28.04	C29:2	♀
8	19.70	C23:1	♀♂	32	28.24	C29:2	♀
9	19.86	C23:1	♀♂	33	28.32	C29:0 br	♀♂
10	20.06	C23:0	♀♂	34	28.41	C29:1	♀
11	21.23	C24:1	♂	35	28.53	C29:1	♀♂
12	21.29	C24:1	♂	36	28.77	C29:0	♀♂
13	21.37	C24:1	♂	37	29.21	C30:0 br	♀♂
14	21.64	C24:0	♂	38	29.33	C30:0 br	♀♂
15	22.47	C25:2	♀♂	39	29.62	C30:1	♂
16	22.62	C25:0 br	♀♂	40	29.79	C30:1	♂
17	22.66	C25:2	♀	41	30.66	C31:2	♀♂
18	22.72	C25:1	♀♂	42	30.87	C31:1 + C31:0br	♀♂
19	22.84	C25:1	♀♂	43	31.00	C31:1	♀♂
20	22.99	C25:1	♀♂	44	31.10	C31:1	♀♂
21	23.16	C25:0	♀♂	45	31.30	C31:0	♀
22	24.12	C26:0 br	♂	46	33.04	C33:2	♀♂
23	25.31	C27:2	♀	47	33.28	C33:1	♀♂
24	25.45	C27:2	♀♂	48	33.34	C33:1	♀♂

### 3.1 Temperature effects over CHC profiles

**Figure 2:**
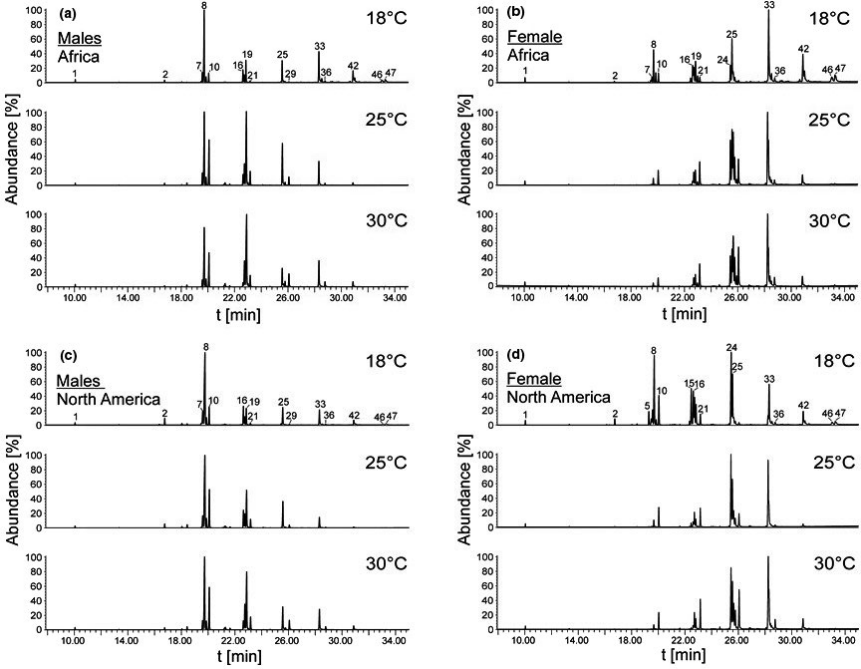
Representative GC chromatograms showing cuticular hydrocarbons (CHCs) of males and females from two continents (Africa and North America) and exposed post‐eclosion to tree different temperatures (18, 25, and 30°C) for 48 hr. The peak numbers indicated correspond to CHCs identifications listed in Table 1

In female samples from Africa, a total of 37 CHCs out of the original 39 passed the 0.5% threshold and were further analyzed. PCA clearly separated three clusters according to the post‐eclosion temperatures (Figure 2a). The 18°C samples appeared more heterogeneous when compared to the remaining two groups that were exposed to 25°C and 30°. CCA with temperature as a categorical predictor showed significant differences among the three groups (Trace = 0.108, *F*‐ratio = 40.362, Monte Carlo test: *p* = .001). Among the variables with the best fit in model predictions, relatively shorter‐chain CHCs (25 carbons or less) and long‐chain CHCs (30 carbons or more) were retrieved as characteristic of the 18°C populations (Figure 2b). In contrast, the relative proportions of medium‐chain lengths CHCs (26–29 carbons) were higher in the 25°C and 30°C post‐eclosion temperature treatments. This temperature‐driven CHC shift is apparent also from the heat map of CHC proportions in Figure 2c. A very similar pattern was observed in female populations from United States. The 37 selected CHCs separated the three populations, with the 18°C population being the most heterogeneous (Figure 2d). CCA confirmed significant differences among treatments using temperature as categorical predictor (Trace = 0.092, *F*‐ratio = 42.472, Monte Carlo test: *p* = .001). Among the variables fitting the best the model predictions, short‐chain (compounds 2, 5–9 and 16) and long‐chain CHCs (38, 43–45, 47 and 48) were retrieved as characteristic of the 18°C populations, while the medium‐chain CHCs were relatively over‐represented in the 25°C and 30°C populations (Figure 2e). The described trend can also be observed in the heat map given in Figure 2f.

**Figure 3:**
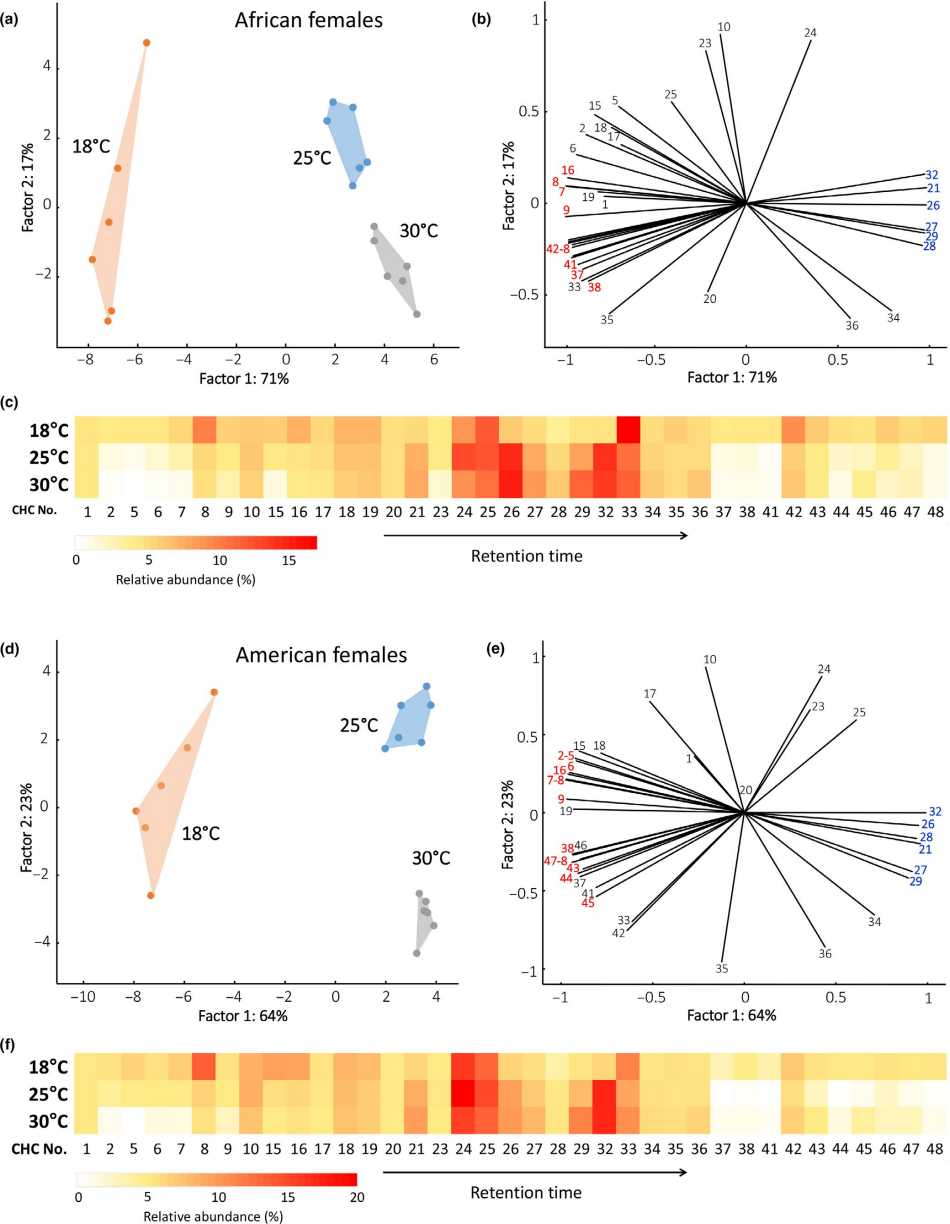
Relative patterns of CHCs in females from Africa and American *D. melanogaster* held at three different post‐eclosion temperatures. (a & d) Projection of PCA factor scores. (b & e). Variable correlations with the first two PCA factors. Variables in bold were retrieved as among the most correlated with the CCA model using temperature as a categorical predictor in the PCA analysis; those in red are over‐represented in 18°C populations, those in blue are characteristic of 25°C and 30°C populations. (c & f) Heat map depicting the relative proportions of the 37 analyzed CHCs in the three groups. Numbering of CHCs corresponds to that in Table 1

Overall, 36 CHCs out of the original 39 were included in the analysis of African male samples. The populations were again well separated based on the post‐eclosion temperatures with the 18°C population being the most diversified (Figure 3a). Temperature was a significant predictor of intergroup differences using CCA (Trace = 0.09, *F*‐ratio = 33.527, Monte Carlo test: *p* = .001). Among the CHCs retrieved to have the best with the model considering temperature as categorical predictor, those with long carbon chains were characteristic of the 18°C populations, while medium‐chain length was relatively more abundant in the 25°C and 30°C populations (Figure 3b). A similar trend can be observed in the heat map showing the relative proportions of the 36 CHCs (Figure 3c) in the populations held at different post‐eclosion temperatures. The US populations of males showed a very similar pattern. The 36 CHCs fitting the inclusion rules were clearly separated by temperature exposure (Figure 3d). Post‐eclosion temperature was a significant categorical predictor using CCA (Trace = 0.051, *F*‐ratio = 29.855, Monte Carlo test: *p* = .001). While very short (compounds 7 and 8) and long‐chain CHCs (compounds 35, 37, 38, 40, 42–44, and 46–48) were retrieved as the most characteristic of the 18°C populations, increase in the relative abundances of medium‐chain CHCs (compounds 10, 12, 14, 18–21 and 29) were correlated with the rising post‐eclosion temperatures, as can also be seen in Figure 3e,f.

### 3.2 Geographical Differences in CHC profiles

**Figure 4:**
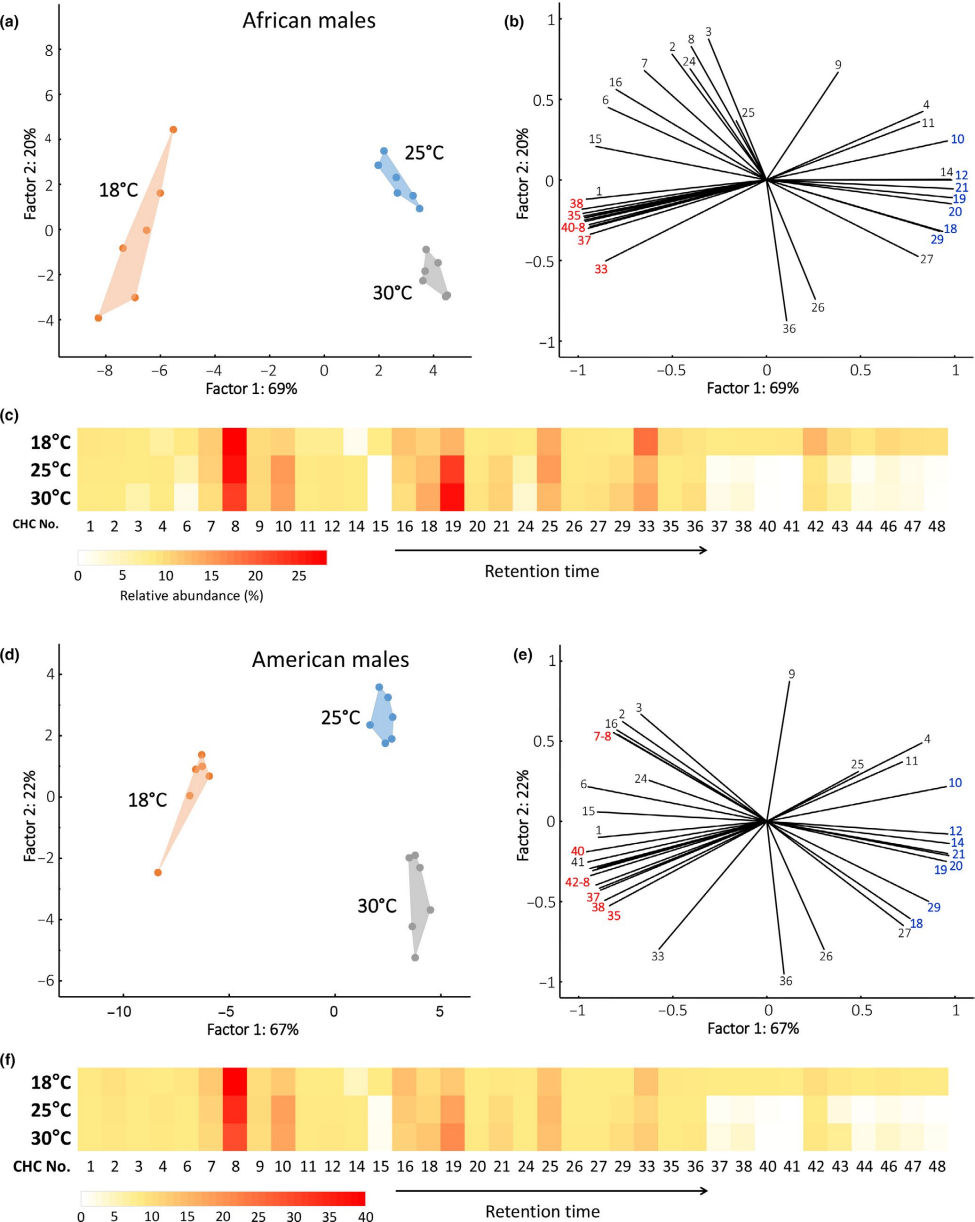
Relative patterns of CHCs in males from African and American *D. melanogaster* populations held at three different post‐eclosion temperatures. (a & d) Projection of PCA factor scores. (b & e) Variable correlations with the first two PCA factors. Variables in bold were retrieved as among the most correlated with the CCA model using temperature as a categorical predictor in the PCA analysis; those in red are over‐represented in 18°C populations, those in blue are characteristic of 25°C and 30°C populations. (c & f) Heat map depicting the relative proportions of the 36 analyzed CHCs in the three groups. Numbering of CHCs corresponds to that in Table 1

In both African as well as in North American populations of *D. melanogaster*, quantitative heterogeneity in CHCs was higher at the lowest assay temperature (18°C). In spite of this apparent variability, our analysis detected significant differences in quantitative profiles between African and American populations for both males and females held at 18°C (Figure 4a, c; CCA *F*‐ratio = 15.81, Monte Carlo test: *p* = .001 for males; *F*‐ratio = 14.21, Monte Carlo test: *p* = .001 for females). In both sexes, the CHCs with lower masses were listed by the CCA analysis as more characteristic of the American population, while the longer chain CHCs were retrieved as over‐represented in the African flies (Figure 4b, d).

### 3.3 Desiccation tolerance and water loss rate (WLR)

**Figure 5:**
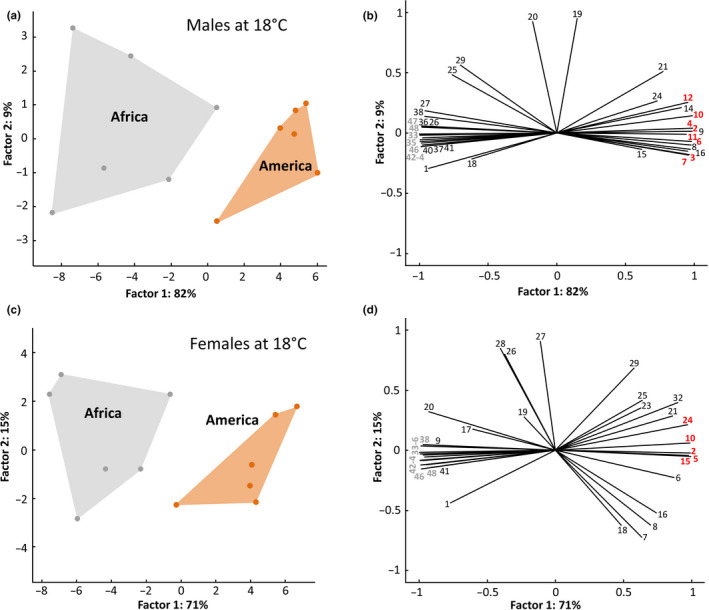
Comparison of quantitative patterns of CHCs between males (a, b) and between females (c, d) from African and American populations, held at 18°C. Numbering of CHCs corresponds to that in Table 1. (a & c) Projection of PCA factor scores. (b & d) Variable correlations with the first two PCA factors. Variables in bold were retrieved as among the most correlated with the CCA model using temperature as a categorical predictor in the PCA analysis (red = American population, gray = African population)

Table 2 depicts ANOVA results for desiccation tolerance where residual values (desiccation LT50 versus. wet mass, dry mass, and water content) were used to rule out any underlying effects due to differences in initial wet mass, dry mass, and/or water content. Post‐eclosion temperature exposure had no effect on desiccation tolerance in either African or North American populations of *D. melanogaster,* but desiccation tolerance was significantly distinct between flies from the two geographic sources (Figure 5; Table 2). As expected, significant sex differences were observed for desiccation tolerance in *D. melanogaster* populations. Sex*temperature infraction terms were also found significant indicating varying sex response to temperature conditions. Three‐way interaction terms (continent*temperature*sex) for wet mass, dry mass, and water content were found significant indicating a complex mechanism behind water regulation in *D. melanogaster* populations. *D. melanogaster* males have higher surface area:volume ratios and they start with less amount of water ( shown as Dry Mass; Table 2).

**Table 2:** ANOVA on desiccation tolerance residuals (calculated against wet mass, dry mass, and water content) for two geographical populations (North America & Africa) of *D. melanogaster* exposed to three temperatures (18°, 25° & 30°C). DT LT_50_: Desiccation tolerance lethal tolerance 50%

Effect	SS	*df*	MS	*F*	*p*
*Wet Mass*					
Continent (Cont)	24.16	1	24.16	5.421	.022*
Temperature (Tem)	11.65	2	5.83	1.308	.276
Sex	1.69	1	1.69	0.379	.54
Cont*Temp	0.35	2	0.17	0.039	.962
Cont*Sex	2.61	1	2.61	0.586	.446
Temp*Sex	47.2	2	23.6	5.296	.007*
Cont*Temp*Sex	56.73	2	28.36	6.364	.003*
Error	374.36	84	4.45		
*Dry Mass*					
Continent (Cont)	38.5	1	38.48	7.338	.008*
Temperature (Tem)	26.8	2	13.38	2.552	.084
Sex	37	1	36.98	7.053	.009*
Cont*Temp	2.1	2	1.04	0.198	.821
Cont*Sex	0	1	0.05	0.009	.923
Temp*Sex	103	2	51.49	9.82	.000*
Cont*Temp*Sex	62.2	2	31.12	5.935	.004*
Error	440.46	84	5.24		
*Water Content*					
Continent (Cont)	23.46	1	23.46	4.959	.029*
Temperature (Tem)	7.9	2	3.95	0.835	.437
Sex	2.83	1	2.83	0.599	.441
Cont*Temp	0.31	2	0.15	0.033	.968
Cont*Sex	4.63	1	4.63	0.978	.326
Temp*Sex	27.09	2	13.55	2.863	.063
Cont*Temp*Sex	56.84	2	28.42	6.007	.004*
Error	397.39	84	4.73		

No significant differences were observed in WLR between African and North American flies (Table 3). However, a marginally nonsignificant *p* value (*p* < .076) was observed for water loss rate due to post‐eclosion temperature treatment (Table 3). As expected, we found significant differences in WLR between sexes.

**Table 3:** ANOVA on water loss rate (nl/hr) for two geographical populations (North America & Africa) of *D. melanogaster* exposed to three temperatures (18°, 25° & 30°C)

Effects	SS	*df*	MS	*F*	*p*
Population (Pop)	60.8	1	60.8	0.494	.483
Temperature (Temp)	644.5	2	322.3	2.617	.076
Sex	2072.3	1	2072.3	16.831	10^–4^*
Pop*Temp	0.1	2	0.0	0.000	.999
Pop*Sex	7.0	1	7.0	0.057	.811
Temp*Sex	521.3	2	260.7	2.117	.123
Pop*Temp*Sex	188.9	2	94.4	0.767	.466
Error	18,838.1	153	123.1		

## 4 DISCUSSION

Holometabolous insect's life cycle generally includes egg, larva, pupa, and adult stage. Once larvae pupate they cannot move until the adult emerges during the process of eclosion. The immobility of the pupae exposes developing adults to a variety of abiotic stresses (primarily temperature) that are experienced during and immediately following eclosion. Many of the biochemical and biophysiological processes associated with cuticle hardening and tanning occur immediately after eclosion (Dewey et al., 2004; Luo et al., 2005). How post‐eclosion environmental temperatures affect adult performance and subsequent fitness is a poorly studied biological phenomenon. CHCs are integral component of insect cuticle, involved in stress resistance and chemical communication (Gibbs & Rajpurohit 2010; Chung & Carroll, 2015). We exposed freshly eclosed adults of *D. melanogaster* (ancestral African and recently migrated North American populations) to three different temperatures (18°, 25°, & 30°C) and examined CHC profiles, desiccation tolerance, and WLR. The results shed light on thermal plasticity of CHCs and physiological associations in an insect model organism.

**Figure 6:**
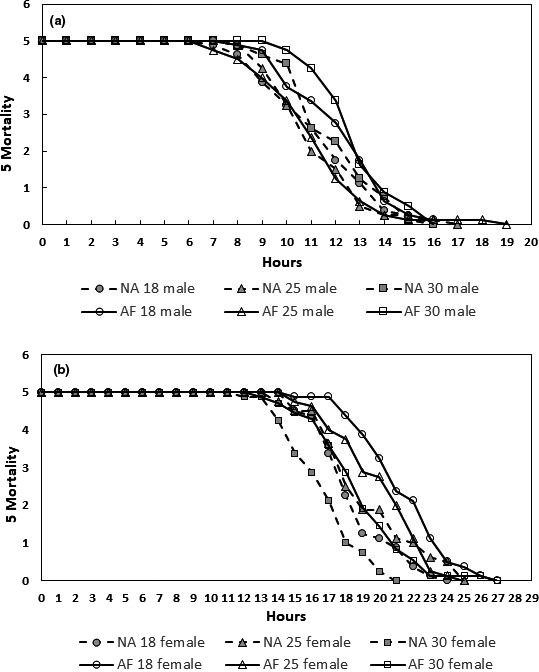
Desiccation tolerance survival curves for males (a) and females (b) for two natural populations of *D. melanogaster* collected from Africa and North America. The assays were run immediately after exposure to three different temperature (18, 25, and 30°C) conditions post‐eclosion for 48 hr. Overall African populations performed better than North American populations under desiccating conditions

A short‐term post‐eclosion exposure to three distinct temperatures (18°, 25°, & 30°C) resulted in a pronounced and comprehensive shift in CHC profiles (Figure 6). Specifically, adults exposed to higher temperatures were characterized by higher concentrations of longer chain length CHCs (Figures 2, 3, &6). There is ample evidence that CHC profiles respond to environmental variation in natural habitats (Toolson & Hadley, 1979; Pardy, 2012; Dembeck et al., 2015; Rajpurohit et al., 2017). CHCs also change seasonally (Rajpurohit et al., 2017; Vander et al., 1989) and more recently it has been established that CHCs evolve over short timescales when temperature changes rapidly and predictably over the growing season (Rajpurohit et al., 2017). Overall, CHCs appear to respond rapidly to variation at multiple scales in natural habitats and might play a key role in response to sudden environmental stress exposure responses in insects.

We found a significant difference in desiccation tolerance between African and North American populations of *D. melanogaster*. *Drosophila* species and populations that are exposed to drier environmental conditions are generally more tolerant to desiccation stress than populations that experience wet or moderate weather conditions (Matzkin et al., 2007). *D. melanogaster* populations living at the higher latitudes of Indian subcontinent are more desiccation tolerant than lower latitude populations (reviewed in Rajpurohit et al., 2013; Rajpurohit & Nedved, 2013). It is still not clear whether “dry‐hot/dry‐cold” versus “humid‐hot/humid‐cold” conditions are more responsible for such changes in desiccation tolerance in *Drosophila* species populations (Rajpurohit et al., 2017). In general Africa is drier and hotter than North America and being more resistant to drought in Africa would be a better adaptive strategy. The ancestral African habitats, that is, Miombo woodlands, are a mix of tropical and subtropical grasslands, savannas, and shrublands. In terms of water availability, these regions get seasonal, that is, wet or dry, but not much difference in temperature (hot versus cold). These are the conditions where water scarcity is much higher, whereas North American habitats are more of a temperature kind of habitats. This could be the reason that African populations are more desiccation tolerant than North American populations.

Free living life stages (adults emerging from pupae) require a variety of activities to survive under natural conditions. Freshly eclosed insects are exposed to variety of abiotic stresses and availability of water is one of them. The physical activities insects perform after eclosion involve breakdown of accumulated resources during larval stages and this process could lower the stored water in the form of various metabolites. Any modification to CHCs would lead to changes in hard cuticle permeability and help in conserving body water. Insects lose > 70% of their body water through the hard cuticle (Chown et al., 2006). Cuticular permeability could change by any modifications in epicuticular hydrocarbon layer and exocuticle layer or in both (Gibbs & Pomonis, 1995; Parkash et al., 2009). Gibbs and Pomonis (1995) suggested that increasing the total amount, saturation, and chain length of epicuticular hydrocarbons could reduce cuticular permeability. Thermal behavior of cuticular hydrocarbons and melanin is complicated and poorly understood (Gibbs & Rajpurohit, 2010; Gibbs, 2011). In a recent work where candidate gene *spidey* expression was inhibited during adulthood, it resulted in loss of oenocyte cells and associated reduction in CHCs production, desiccation tolerance, and life span (Chiang et al., 2016). Temperature relations of such regulators have not been studied yet.

Whether thermally mediated changes to post‐eclosion adult CHCs account for water loss changes will causatively shed light on the adaptive aspects of this interrelationship. It was surprising to note that there was no significant difference observed in water loss rate between African and North American populations of *D. melanogaster*. Rajpurohit et al. (2017) found similar patterns where endpoint populations of a cline (along the east coast of United States) showed differences in desiccation tolerance which was not associated with water loss rate. In their study, males showed a varying metabolic rate under prolonged desiccation, indicating behavioral adjustments at the organismal level (Rajpurohit et al., 2017). It is clearly evidenced that CHCs are multifunctional molecules (involved in stress response, sexual selection, and communication), further work is needed to make causative connections.

## 5 CONCLUSIONS

Taken together, these findings demonstrate that CHC profiles exhibit a rapid and plastic response to post‐eclosion temperature and that this response varies between African and North American populations. These observed differences in CHC profiles are also associated with plasticity, and geographic differentiation, in desiccation tolerance. Surprisingly, however, variation in CHC profiles and desiccation tolerance were not associated with differences in water loss rate. In insects water balance mechanisms are complicated and it could occur through many ways, that is, by increasing the body water content and tolerating greater amounts of water loss besides reducing the rate of water loss (Gibbs et al., 1997; Gibbs & Matzkin, 2001; Hoffmann & Parsons, 1989). Our study first time shed light on early age temperature effects on organismal fitness. This work also emphasizes holometabolous insects life under varying environmental conditions that while addressing stress response issues crucial transitions phases from nonmotile life stages (i.e., pupal stages) to free life stages (as adults).

## CONFLICTS OF INTEREST

None declared.

## AUTHOR CONTRIBUTION

**Subhash Rajpurohit:** Conceptualization (equal); Data curation (equal); Formal analysis (equal); Funding acquisition (equal); Investigation (equal); Methodology (equal); Project administration (equal); Resources (equal); Supervision (lead); Writing‐original draft (equal); Writing‐review & editing (equal). **Vladimir Vrkoslav:** Data curation (equal); Formal analysis (equal); Writing‐review & editing (equal). **Robert Hanus:** Formal analysis (equal); Software (equal); Visualization (equal); Writing‐review & editing (equal). **Allen Gibbs:** Formal analysis (equal); Funding acquisition (equal); Methodology (equal); Resources (equal); Writing‐review & editing (equal). **Josef Cvacka:** Funding acquisition (equal); Methodology (equal); Resources (equal); Writing‐review & editing (equal). **Paul S. Schmidt:** Conceptualization (equal); Formal analysis (equal); Funding acquisition (equal); Project administration (equal); Resources (equal); Supervision (equal); Writing‐review & editing (equal).

## Data Availability

All data are archived at Dryad doi.org/10.5061/dryad.1g1jwsttk.
